# Conditional Rényi Divergence Saddlepoint and the Maximization of *α*-Mutual Information

**DOI:** 10.3390/e21100969

**Published:** 2019-10-04

**Authors:** Changxiao Cai, Sergio Verdú

**Affiliations:** 1 Department of Electrical Engineering, Princeton University, C307 Engineering Quadrangle, NJ 08540, USA; ccai@princeton.edu; 2 Independent Researcher, Princeton, NJ 08540, USA

**Keywords:** information measures, relative entropy, conditional relative entropy, mutual information, Rényi divergence, α-mutual information, channel capacity, minimax redundancy

## Abstract

Rényi-type generalizations of entropy, relative entropy and mutual information have found numerous applications throughout information theory and beyond. While there is consensus that the ways A. Rényi generalized entropy and relative entropy in 1961 are the “right” ones, several candidates have been put forth as possible mutual informations of order α. In this paper we lend further evidence to the notion that a Bayesian measure of statistical distinctness introduced by R. Sibson in 1969 (closely related to Gallager’s $E_{0}$ function) is the most natural generalization, lending itself to explicit computation and maximization, as well as closed-form formulas. This paper considers general (not necessarily discrete) alphabets and extends the major analytical results on the saddle-point and saddle-level of the conditional relative entropy to the conditional Rényi divergence. Several examples illustrate the main application of these results, namely, the maximization of α-mutual information with and without constraints.

## 1. Introduction

The Rényi divergence of order α between two probability measures defined on the same measurable space,

(1) $$\begin{array}{c} D_{\alpha }\left(P\;∥\;Q\right)=\frac{1}{\alpha -1}\log\int {\left(\frac{dP}{dQ}\left(x\right)\right)}^{\alpha }\;dQ\left(x\right), \end{array}$$

is a useful generalization of the relative entropy D(P∥Q) introduced by Rényi [1] in the discrete case $(\lim_{\alpha ↑1}D_{\alpha }\left(P\;∥\;Q\right)=D\left(P∥Q\right)$). Many of the properties satisfied by relative entropy hold for Rényi divergence, such as nonnegativity, convexity, lower semicontinuity, data processing inequality, and additivity for product measures. $D_{\alpha }\left(P∥Q\right)$ can be defined in more generality without requiring P≪Q. A comprehensive survey of the properties satisfied by Rényi divergence can be found in [2]. Just as D(P∥Q), $D_{\alpha }\left(P\;∥\;Q\right)$ provides a useful gauge of the distinctness of *P* and *Q*, which has found applications in large deviations problems (such as the asymptotic analysis of hypothesis testing [3,4,5]), lossless data compression [4,6,7], data transmission through noisy channels [8,9,10], and statistical physics [11]. If $P_{1}\ll P_{0}$, then Rényi divergence of order α∈(0,1)∪(1,∞) can be expressed in terms of relative entropy through [5]

(2) $$\begin{array}{c} \left(1-\alpha \right)D_{\alpha }\left(P_{1}∥P_{0}\right)=\min_{P\ll P_{1}}\left\{\alpha \;D\left(P∥P_{1}\right)+\left(1-\alpha \right)\;D\left(P∥P_{0}\right)\right\}. \end{array}$$

Although not an *f*-divergence, there is a one-to-one correspondence between Rényi divergence and Hellinger divergence $H_{\alpha }\left(P∥Q\right)$ (e.g., [12])

(3) $$\begin{array}{c} D_{\alpha }\left(P∥Q\right)=\frac{1}{\alpha -1}\log\left(1+\left(\alpha -1\right)H_{\alpha }\left(P∥Q\right)\right). \end{array}$$

One of the major applications of relative entropy is to quantify statistical dependence in a joint probability measure by means of the mutual information

(4) $$\begin{array}{c} I\left(X;Y\right)=D\left(P_{XY}\;∥\;P_{X}\times P_{Y}\right). \end{array}$$

The corresponding straight generalization replacing relative entropy by Rényi divergence is also a measure of dependence but has found scant utility so far (see [6,13]). To explore the generalization that we study in this paper, namely α-mutual information, we need to consider the conditional versions of relative entropy and Rényi divergence. These are defined in general for two random transformations $P_{Y|X}$ and $Q_{Y|X}$ and an unconditional probability measure $P_{X}$ simply as

(5) $$\begin{array}{cc} D(P_{Y|X}\;∥\;Q_{Y|X}|P_{X}) & =D(P_{Y|X}P_{X}∥Q_{Y|X}P_{X}), \end{array}$$

(6) $$\begin{array}{cc} D_{\alpha }(P_{Y|X}\;∥\;Q_{Y|X}\left|P_{X}\right) & =D_{\alpha }\left(P_{Y|X}P_{X}∥Q_{Y|X}P_{X}\right). \end{array}$$

A major difference between those conditional measures is that while $D(P_{Y|X}\;∥\;Q_{Y|X}|P_{X})$ is plainly the expectation $\int D\left(P_{Y|X=x}∥\;Q_{Y|X=x}\right)\;dP_{X}\left(x\right)$, the conditional Rényi divergence depends on the function $D_{\alpha }\left(P_{Y|X=x}∥\;Q_{Y|X=x}\right)$ in a more involved way. In this paper, the use of the conditional information measures will be circumscribed to the special case in which $Q_{Y|X}$ is actually an unconditional measure. In fact, a more productive way to express mutual information than (4) is the asymmetric expression

(7) $$\begin{array}{cc} I(X;Y) & =D(P_{Y|X}\;∥\;P_{Y}|P_{X}) \end{array}$$

(8) $$\begin{array}{cc} \; & =\min_{Q}D(P_{Y|X}\;∥\;Q\;|\;P_{X}). \end{array}$$

Equation (8) follows from the key additive decomposition formula

(9) $$\begin{array}{c} D(P_{Y|X}∥P_{Y}|P_{X})=D(P_{Y|X}∥Q_{Y}\left|P_{X}\right)-D\left(P_{Y}∥Q_{Y}\right), \end{array}$$

where $Q_{Y}$ is an arbitrary measure dominating $P_{Y}$. We see that (8) is a Bayesian measure of the distinctness of the constellation of probability measures $\left\{P_{Y|X=x},x\in A\right\}$, sometimes referred to as *information radius*, where the center of gravity of the constellation is none other than $P_{Y}$. Equation (8) has proven to be very fertile, particularly when it comes to supremize I(X;Y) with respect to $P_{X}$ since the ensuing supmin optimization has a saddle-point if and only if there is an input distribution that attains the maximal mutual information. The convexity of $D(P_{Y|X}\;∥\;Q\;|\;P_{X})$ in *Q* and concavity (linearity) in $P_{X}$, along with the minimax theorem ensures the existence of the saddle-point whenever the set of allowed input distributions is compact. The Arimoto-Blahut algorithm [14,15] for finding maxI(X;Y) in finite alphabet settings is also inspired by (8).

Mutual information $I\left(X;Y\right)=I\left(P_{X},P_{Y|X}\right)$ also possesses a saddle point (assuming convexity and compactness of the corresponding feasible sets) since it is concave in $P_{X}$ (to see that, nothing better than (9)) and is convex in $P_{Y|X}$. This property has found rich applications in information theory (e.g., [16,17,18]) but neither it nor its generalization to α-mutual information will not concern us in this paper.

Even if a saddle-point for the conditional relative entropy does not exist, Kemperman [19] showed that supmin can be swapped, thereby establishing the existence of a *saddle value*.

Another well-known application of (8) and the conditional relative entropy saddle-point is the so-called *channel capacity-minimax redundancy* theorem due to Gallager [20] and Ryabko [21] (see also [22,23]), which shows that the maximal mutual information obtained with a finite constellation $\left\{P_{Y|X=x},x\in A\right\}$ is equal to the minimax redundancy in universal lossless data compression of an unknown source selected from $\left\{P_{Y|X=x},x\in A\right\}$. Notable generalizations of this result to infinite alphabets without requiring that a distribution maximizing mutual information exists are due to Kemperman [19] and Haussler [24]. Recently, the Rényi counterpart of the channel capacity-minimax redundancy result has also been considered, under various restrictions, in [2,25,26].

The main purpose of this paper is to generalize the saddle-point property of conditional relative entropy and its applications to the maximization of mutual information when relative entropy is replaced by Rényi divergence. Towards that end, we recall the various directions in which mutual information has been generalized using Rényi divergence (see also [27]):

1. As aforementioned, the straight generalization $D_{\alpha }\left(P_{XY}\;∥\;P_{X}\times P_{Y}\right)$ has not yet found wide applicability.
2. In the discrete case and α∈(0,1)∪(1,∞), Arimoto [28] proposed the definition of the nonnegative quantity
  (10) $$\begin{array}{c} I^{a}\left(X;Y\right)=H_{\alpha }\left(X\right)-H_{\alpha }^{a}\left(X|Y\right), \end{array}$$
  where the Rényi entropy [1] and Arimoto-Rényi conditional entropy [28] are
  (11) $$\begin{array}{cc} H_{\alpha }\left(X\right) & =\frac{\alpha }{1-\alpha }\log{∥P_{X}∥}_{\alpha }, \end{array}$$
  (12) $$\begin{array}{cc} H_{\alpha }^{a}\left(X|Y\right) & =\frac{\alpha }{1-\alpha }\log E[∥P_{X|Y}\left(\cdot |Y\right){∥}_{\alpha }] \end{array}$$
  with the α-norm of a probability mass function denoted as ${∥P∥}_{\alpha }={\left(\sum_{x\in A}P^{\alpha }\left(x\right)\right)}^{\frac{1}{\alpha }}$. Arimoto extended his algorithm in [14] to compute what he called the *capacity of order* α,
  (13) $$\begin{array}{c} C_{\alpha }^{a}=\max_{X}I^{a}\left(X;Y\right), \end{array}$$
  for finite-alphabet random transformations and showed that there exist codes of rate *R* and blocklength *n* whose error probability is upper bounded by
  $$\underset{\alpha \in (\frac{1}{2},1)}{\inf}\exp\left(\frac{\alpha -1}{\alpha }\left(C_{\alpha }^{a}-R\right)\right).$$
3. Augustin [29] and, later, Csiszár [4] defined
  (14) $$\begin{array}{c} I_{\alpha }^{c}\left(X;Y\right)=\min_{Q_{Y}}E\left[D_{\alpha }\left(P_{Y|X}\left(\cdot |X\right)\;∥\;Q_{Y}\right)\right]. \end{array}$$
  $C_{\alpha }^{c}=\max_{X}I_{\alpha }^{c}\left(X;Y\right)$ is dubbed the *Augustin capacity of order* α in [30]. Csiszár [4] showed that for $\alpha \in (\frac{1}{2},1)$, $I_{\alpha }^{c}\left(X;Y\right)$ is the intercept on the *R*-axis of a supporting line of slope $1-\frac{1}{\alpha }$ of the error exponent function for codes of rate *R* with constant-composition $P_{X}$. Unfortunately, the minimization in (14) is not amenable to explicit solution.
4. For the purpose of coming up with a measure of the similarity among a finite collection of probability measures $\left\{P_{Y|X=x},x\in A\right\}$, weighted by $P_{X}$ on A, Sibson [31] proposed the *information radius of order* α as
  (15) $$\begin{array}{c} I_{\alpha }\left(X;Y\right)=\min_{Q}D_{\alpha }(P_{Y|X}\;∥\;Q|P_{X}). \end{array}$$
  As we will see, the minimization in (15) can be solved explicitly. This is the generalization of mutual information we adopt in this paper and which, as in [27], we refer to as α-mutual information. A word of caution is that in [4], the symbols $I_{\alpha }\left(X;Y\right)$ and $K_{\alpha }\left(X;Y\right)$ are used in lieu of what we denote $I_{\alpha }^{c}\left(X;Y\right)$ and $I_{\alpha }\left(X;Y\right)$, respectively. $C_{\alpha }=\max_{X}I_{\alpha }\left(X;Y\right)$ is dubbed the *Rényi capacity of order* α in [26].
5. Independently, Lapidoth-Pfister [32] and Tomamichel-Hayashi [33] proposed
  (16) $$\begin{array}{c} I_{\alpha }^{l}\left(X;Y\right)=\min_{Q_{X}}\min_{Q_{Y}}D_{\alpha }\left(P_{XY}\;∥\;Q_{X}\times Q_{Y}\right). \end{array}$$
  and showed that it determines the performance of composite hypothesis tests for independence where the hypothesized joint distribution is known but under the independence hypothesis the marginals are unknown. It was shown in [34] that
  (17) $$\begin{array}{c} I_{\alpha }^{c}\left(X;Y\right)\leq I_{\alpha }^{l}\left(X;Y\right)\leq I_{\alpha }\left(X;Y\right). \end{array}$$

Despite the difference in the definitions of the various versions, it was shown in the discrete setting that [4,28].

(18) $$\begin{array}{c} C_{\alpha }^{a}=C_{\alpha }^{c}=C_{\alpha } \end{array}$$

Therefore, solving for $\max_{X}I_{\alpha }\left(X;Y\right)$ carries added significance, whenever one of the other definitions is adopted. Note that (17) and (18) imply that $C_{\alpha }=\max_{P_{X}}I_{\alpha }^{l}\left(P_{X},P_{Y|X}\right)$. A major application for the maximization of $I_{\alpha }\left(X;Y\right)$ is in the large deviation analysis of optimal data transmission codes since the sphere-packing error exponent function and the random-coding error exponent function

(19) $$\begin{array}{cc} E_{\mathrm{sp}}\left(R\right) & =\underset{\rho \geq 0}{\sup}\left\{\rho \;C_{\frac{1}{1+\rho }}-\rho \;R\right\}, \end{array}$$

(20) $$\begin{array}{cc} E_{r}\left(R\right) & =\underset{\rho \in [0,1]}{\sup}\left\{\rho \;C_{\frac{1}{1+\rho }}-\rho \;R\right\}, \end{array}$$

popularized in [35] and [36], respectively, are upper and lower bounds to the channel reliability function, respectively. A function similar to (20) has recently been shown [37] to yield the large deviations behavior of random coding in the setting of channel resolvability.

The organization of the paper is as follows. Section 2 states the definitions and properties of the various information measures that are used throughout the paper. In particular, we introduce the key notion of α-response to an input probability measure through a given random transformation. In Section 3 we present the main results (with proofs relegated to Section 5) related to the saddle-point and saddle-value of the conditional Rényi divergence, allowing the optimization to be circumscribed to any convex set of input probability measures. The equivalence of the existence of a probability measure that maximizes α-mutual information and the existence of a saddle point is shown and several illustrative examples of the use of this result in the computation of $C_{\alpha }$ are also given. The fact that a saddle-level exists (i.e., supmin commute) even if there is no input probability measure that achieves the supremum α-mutual information is established, thereby generalizing Kemperman’s [19] saddle-level result to Rényi divergence through a different route than that followed in [26].

## 2. Notation, Definitions and Properties

1. If (A,F,P) is a probability space, X∼P indicates P[X∈F]=P(F) for all F∈F.
2. Let (A,F) and (B,G) be measurable spaces, which we refer to as the input and output spaces, respectively, with A and B referred to as the input and output *alphabets* respectively. $P_{Y|X}:A\to B$ denotes a *random transformation* from A to B, i.e., for any x∈A, $P_{Y|X=x}\left(\cdot \right)$ is a probability measure on (B,G), and for any B∈G, $P_{Y|X=\cdot }\left(B\right)$ is an F-measurable function. For brevity, we will usually drop mention of the underlying σ-fields. If *P* is a probability measure on A and $P_{Y|X}:A\to B$ is a random transformation, the corresponding joint probability measure on A×B is denoted by $P\;P_{Y|X}$ (or, interchangeably, $P_{Y|X}P$). The notation $P\to P_{Y|X}\to Q$ indicates that the output marginal of the joint probability measure $P\;P_{Y|X}$ is denoted by *Q*.
3. The *relative information* ${ı}_{P∥Q}\left(x\right)$ between two probability measures *P* and *Q* on the same measurable space such that P≪Q is defined as
  (21) $$\begin{array}{c} {ı}_{P∥Q}\left(x\right)=\log\frac{\;dP}{\;dQ}\left(x\right), \end{array}$$
  where $\frac{\;dP}{\;dQ}$ is the Radon-Nikodym derivative of *P* with respect to *Q*. The *relative entropy* is
  (22) $$\begin{array}{c} D\left(P∥Q\right)=E[{ı}_{P_{X}∥Q_{X}}\left(X\right)],\;X∼P. \end{array}$$
4. Given $P_{X}\to P_{Y|X}\to P_{Y}$, the *information density* is defined as
  (23) $$\begin{array}{c} {ı}_{X;Y}\left(a;b\right)={ı}_{P_{Y|X=a}∥P_{Y}}\left(b\right),\;\left(a,b\right)\in A\times B. \end{array}$$
5. Fix α>0, $P_{Y|X}:A\to B$, and a probability measure $P_{X}$ on A. Then, the output probability measure $P_{Y_{\left[\alpha \right]}}$ is called the α-*response* to $P_{X}$ if
  (24) $$\begin{array}{c} {ı}_{Y_{\left[\alpha \right]}∥Y}\left(y\right)=\frac{1}{\alpha }\log E\left[\exp\left(\alpha \;{ı}_{X;Y}\left(X;y\right)-\kappa_{\alpha }\right)\right],\;X∼P_{X}, \end{array}$$
  where $P_{X}\to P_{Y|X}\to P_{Y}$, and $\kappa_{\alpha }$ is a scalar that guarantees that $P_{Y_{\left[\alpha \right]}}$ is a probability measure. For notational convenience, we omit the dependence of $\kappa_{\alpha }$ on $P_{X}$ and $P_{Y|X}$. Equivalently, if $p_{Y_{\left[\alpha \right]}}$ and $p_{Y|X}$ denote the densities with respect to some dominating measure, then (24) becomes
  (25) $$\begin{array}{c} p_{Y_{\left[\alpha \right]}}\left(y\right)=\exp\left(-\frac{\kappa_{\alpha }}{\alpha }\right)E^{\frac{1}{\alpha }}\left[p_{Y|X}^{\alpha }\left(y|X\right)\right],\;X∼P_{X}. \end{array}$$
  In particular, the 1-*response* to $P_{X}$ is $P_{Y}$. In [26], the α-response to $P_{X}$ is dubbed the order α *Rényi mean for prior* $P_{X}$.
6. Given two probability measures *P* and *Q* on the same measurable space and a scalar α∈(0,1)∪(1,∞), the *Rényi divergence* of order α between *P* and *Q* is defined as [1]
  (26) $$\begin{array}{c} D_{\alpha }\left(P∥Q\right)=\frac{1}{\alpha -1}\log\int_{A}p^{\alpha }q^{1-\alpha }\;d\mu , \end{array}$$
  where *p* and *q* are the Radon-Nikodym derivatives of *P* and *Q*, respectively, with respect to a common dominating σ-finite measure μ. We define $D_{1}\left(P∥Q\right)=D\left(P∥Q\right)$ as this coincides with the limit from the left at α=1. It is also the limit from the right whenever $D_{\alpha }\left(P∥Q\right)<\infty$ for some α>1. The cases α=0 and α=∞ can be defined by taking the corresponding limits. In this work, we only focus on the simple orders of α, i.e., α∈(0,1)∪(1,∞). As we saw in (1), if P≪Q, then (26) becomes
  (27) $$\begin{array}{cc} D_{\alpha }\left(P\;∥\;Q\right) & =\frac{1}{\alpha -1}\log\left(E\left[\exp\left(\alpha \;{ı}_{P∥Q}\left(W\right)\right)\right]\right),\;W∼Q \end{array}$$
  (28) $$\begin{array}{cc} \; & =\frac{1}{\alpha -1}\log\left(E\left[\exp\left(\left(\alpha -1\right)\;{ı}_{P∥Q}\left(V\right)\right)\right]\right),\;V∼P \end{array}$$
7. If α∈(0,1)∪(1,∞), then the binary Rényi divergence of order α is given by
  (29) $$\begin{array}{cc} d_{\alpha }\left(p∥q\right) & =D_{\alpha }\left(\left[p\;\;1-p\right]∥\left[q\;\;1-q\right]\right) \end{array}$$
  (30) $$\begin{array}{cc} \; & =\frac{1}{\alpha -1}\log\left(p^{\alpha }q^{1-\alpha }+{\left(1-p\right)}^{\alpha }{\left(1-q\right)}^{1-\alpha }\right). \end{array}$$
  Note that
  (31) $$\begin{array}{c} \lim_{\alpha \to 1}d_{\alpha }\left(p∥\frac{1}{2}\right)=\log2-h\left(p\right), \end{array}$$
  where the usual binary entropy function is denoted by $h\left(x\right)=x\log\frac{1}{x}+\left(1-x\right)\log\frac{1}{1-x}$. Given $\left(p_{0},p_{1}\right)\in {\left(0,1\right)}^{2}$, $p_{0}\neq p_{1}$, the solution to $d_{\alpha }\left(p_{0}∥q\right)=d_{\alpha }\left(p_{1}∥q\right)$ is
  (32) $$\begin{array}{c} q={\left(1+{\left(\frac{p_{0}^{\alpha }-p_{1}^{\alpha }}{{\left(1-p_{1}\right)}^{\alpha }-{\left(1-p_{0}\right)}^{\alpha }}\right)}^{\frac{1}{1-\alpha }}\right)}^{-1}. \end{array}$$
8. $D_{\alpha }\left(P\;∥\;Q\right)\geq 0$, with equality only if P=Q.
9. $D_{\alpha }\left(P\;∥\;Q\right)$ is monotonically increasing with α.
10. While we may have D(P∥Q)=∞ and P≪Q simultaneously, $D_{\alpha }\left(P∥Q\right)=\infty$ for any α∈(0,1) is equivalent to *P* and *Q* being orthogonal. Conversely, if for some α>1, $D_{\alpha }\left(P∥Q\right)<\infty$, then P≪Q.
11. The Rényi divergence satisfies the *data-processing inequality*. If $P_{X}\to P_{Y|X}\to P_{Y}$ and $Q_{X}\to P_{Y|X}\to Q_{Y}$, then
  (33) $$\begin{array}{c} D_{\alpha }\left(P_{X}∥Q_{X}\right)\geq D_{\alpha }\left(P_{Y}∥Q_{Y}\right). \end{array}$$
12. Gilardoni [38] gave a strengthened Pinsker’s inequality upper bounding the square of the total variation distance by
  (34) $$\begin{array}{cc} {\left|P-Q\right|}^{2} & \leq \underset{\alpha \in (0,1]}{\inf}\frac{1}{2\alpha }D_{\alpha }\left(P∥Q\right) \end{array}$$
  (35) $$\begin{array}{cc} \; & \leq \underset{\alpha >0}{\inf}\frac{1}{2\min\{\alpha ,1\}}D_{\alpha }\left(P∥Q\right), \end{array}$$
  where we have used the monotonicity in α of the Rényi divergence.
13. The Rényi divergence is lower semicontinuous in the topology of setwise convergence, i.e., if for every event A∈F, $P_{n}\left(A\right)\to P\left(A\right)$, and $Q_{n}\left(A\right)\to Q\left(A\right)$, then
  (36) $$\begin{array}{c} \underset{n\to \infty }{lim inf}D_{\alpha }\left(P_{n}∥Q_{n}\right)\geq D_{\alpha }\left(P∥Q\right),\;\alpha \in \left(0,\infty \right]. \end{array}$$
  In particular, note that (36) holds if $|P_{n}-Q_{n}|\to 0$.
14. In the theory of robust lossless source coding [22,25] the following scalar, called the α-*minimax redundancy* of $P_{Y|X}$, is an important measure of the worst-case redundancy penalty that ensues when the encoder only knows that the data is generated according to one of the probability measures in the collection $\left\{P_{Y|X=x},x\in A\right\}$:
  (37) $$\begin{array}{c} R_{\alpha }=\underset{Q_{Y}}{\inf}\underset{x\in A}{\sup}D_{\alpha }\left(P_{Y|X=x}∥\;Q_{Y}\right), \end{array}$$
  where the infimum is over all the probability measures on B.
15. Given input distribution $P_{X}$ and random transformations $P_{Y|X},Q_{Y|X}$, the *conditional Rényi divergence* of order α∈(0,1)∪(1,∞) is
  (38) $$\begin{array}{cc} D_{\alpha }(P_{Y|X}\;∥\;Q_{Y|X}\left|P_{X}\right) & =D_{\alpha }\left(P_{X}P_{Y|X}\;∥\;P_{X}Q_{Y|X}\right). \end{array}$$
  Although (38) also holds for the familiar α=1 case, in general the conditional Rényi divergence is *not* the arithmetic average of $D(P_{Y|X=x}∥Q_{Y})$ with respect to $P_{X}$ if α≠1. Instead it’s a generalized mean, or a scaled cumulant generating function evaluated at α−1. Specifically, if $X∼P_{X}$, then
  (39) $$\begin{array}{cc} D_{\alpha }(P_{Y|X}∥Q_{Y}\left|P_{X}\right) & =\frac{1}{\alpha -1}\log E\left[\exp\left(\left(\alpha -1\right)D_{\alpha }\left(P_{Y|X}\left(\cdot |X\right)∥Q_{Y}\right)\right)\right]. \end{array}$$
  Regardless of whether α∈(0,1) or α∈(1,∞), (39) implies that
  (40) $$\begin{array}{cc} D_{\alpha }(P_{Y|X}∥Q_{Y}\left|P_{X}\right) & \leq \underset{x\in A}{\sup}D_{\alpha }\left(P_{Y|X=x}∥Q_{Y}\right) \end{array}$$
  (41) $$\begin{array}{cc} \; & =\underset{P_{X}}{\sup}D_{\alpha }(P_{Y|X}∥Q_{Y}\left|P_{X}\right) \end{array}$$
  with the supremum in (41) over all input probability measures.
16. The key additive decomposition formula for the mutual information (9) has a nice counterpart for the α-mutual information [27]. Let $P_{X}\to P_{Y|X}\to P_{Y}$ and $Q_{Y}$ be an arbitrary probability measure on B such that $P_{Y}\ll Q_{Y}$. Then, it is easy to verify that
  (42) $$\begin{array}{c} D_{\alpha }(P_{Y|X}∥P_{Y_{\left[\alpha \right]}}|P_{X})=D_{\alpha }(P_{Y|X}∥Q_{Y}\left|P_{X}\right)-D_{\alpha }\left(P_{Y_{\left[\alpha \right]}}∥Q_{Y}\right), \end{array}$$
  a relationship noted by Sibson [31] in the discrete case.
17. Given α>0, $P_{X}$ and $P_{Y|X}$, the *α-mutual information* is [27,31]
  (43) $$\begin{array}{cc} I_{\alpha }\left(X;Y\right) & =\min_{Q}D_{\alpha }(P_{Y|X}\;∥\;Q|P_{X}) \end{array}$$
  (44) $$\begin{array}{cc} \; & =D_{\alpha }(P_{Y|X}\;∥\;P_{Y_{\left[\alpha \right]}}\left|P_{X}\right) \end{array}$$
  (45) $$\begin{array}{cc} \; & =\frac{1}{\alpha -1}\log E\left[\exp\left(\left(\alpha -1\right)D_{\alpha }\left(P_{Y|X}\left(\cdot |X\right)∥P_{Y_{\left[\alpha \right]}}\right)\right)\right] \end{array}$$
  (46) $$\begin{array}{cc} \; & =D_{\alpha }(P_{Y|X}\;∥\;P_{Y}\left|P_{X}\right)-D_{\alpha }\left(P_{Y_{\left[\alpha \right]}}\;∥\;P_{Y}\right), \end{array}$$
  where $P_{X}\to P_{Y|X}\to P_{Y}$. It can be checked that the constant in (24) is equal to
  (47) $$\begin{array}{cc} \kappa_{\alpha } & =\left(\alpha -1\right)I_{\alpha }\left(X;Y\right). \end{array}$$
  Note that $I_{1}\left(X;Y\right)=I\left(X;Y\right)$ but, in general, $I_{\alpha }\left(X;Y\right)\neq I_{\alpha }\left(Y;X\right)$.
18. An alternative expression for α-mutual information, which will come in handy in our analysis and which does not involve either $P_{Y}$ or $P_{Y_{\left[\alpha \right]}}$ is obtained by introducing an auxiliary probability measure $P_{\bar{Y}}$ dominating the collection $\left\{P_{Y|X=u},u\in A\right\}$ [27]:
  (48) $$\begin{array}{cc} I_{\alpha }\left(X;Y\right) & =\frac{\alpha }{\alpha -1}\log E\left[E^{\frac{1}{\alpha }}\left[\exp\left(\alpha \;{ı}_{X;\bar{Y}}\left(X;\bar{Y}\right)\right)|\bar{Y}\right]\right],\;\left(X,\bar{Y}\right)∼P_{X}\times P_{\bar{Y}}, \end{array}$$
  where
  (49) $$\begin{array}{c} {ı}_{X;\bar{Y}}\left(x;y\right)=\log\frac{dP_{Y|X=x}}{dP_{\bar{Y}}}\left(y\right). \end{array}$$
  As usual, sometimes it is convenient to fix σ-finite measures $\mu_{X}$ and $\mu_{Y}$ on the input and output spaces which dominate $P_{X}$ and $\left\{P_{Y|X=x}:x\in A\right\}$, respectively, and denote their densities with respect to the reference measures by
  (50) $$\begin{array}{cc} p_{Y|X}\left(y|x\right) & =\frac{\;dP_{Y|X=x}}{\;d\mu_{Y}}\left(y\right), \end{array}$$
  (51) $$\begin{array}{cc} p_{X}\left(x\right) & =\frac{\;dP_{X}}{\;d\mu_{X}}\left(x\right). \end{array}$$
  Then, we can write α-mutual information as
  (52) $$\begin{array}{cc} I_{\alpha }\left(P_{X},P_{Y|X}\right) & =\frac{\alpha }{\alpha -1}\log\int_{B}{\left(\int_{A}p_{Y|X}^{\alpha }\left(y|x\right)p_{X}\left(x\right)\;d\mu_{X}\left(x\right)\right)}^{\frac{1}{\alpha }}\;d\mu_{Y}\left(y\right). \end{array}$$
19. In the special case of discrete alphabets,
  (53) $$\begin{array}{c} E_{0}\left(\rho ,P_{X},P_{Y|X}\right)=\rho \;I_{\frac{1}{1+\rho }}\left(X;Y\right), \end{array}$$
  where the left side is the familiar Gallager function defined in [36] for ρ∈(0,1) as
  (54) $$\begin{array}{c} E_{0}\left(\rho ,P_{X},P_{Y|X}\right)=-\log\sum_{y\in B}{\left(\sum_{x\in A}P_{X}\left(x\right)P_{Y|X}^{\frac{1}{1+\rho }}\left(y|x\right)\right)}^{1+\rho }. \end{array}$$
20. Fix α>0, $P_{Y|X}:A\to B$, and a collection P of probability measures on the input space. Then, we denote
  (55) $$\begin{array}{c} C_{\alpha }\left(P\right)=\underset{P_{X}\in P}{\sup}I_{\alpha }\left(P_{X},P_{Y|X}\right). \end{array}$$
  When P is the set of all input measures we write simply $C_{\alpha }$, dubbed the *Rényi capacity* in [26]. $C_{\alpha }$ is a measure of the similarity of the family $\left\{P_{Y|X=x},x\in A\right\}$, which plays an important role in the analysis of the fundamental limits of information transmission through noisy channels, particularly in the regime of exponentially small error probability. For a long time (e.g., [39]) the *cutoff rate* $C_{\frac{1}{2}}$ was conjectured to be the maximal rate for which reliable codes with manageable decoding complexity can be found. The zero-error capacity of the discrete memoryless channel with feedback is equal to either zero or [40]
  (56) $$\begin{array}{cc} C_{0f} & =\max_{X}I_{0}\left(X;Y\right), \end{array}$$
  depending on whether there is $\left(a_{1},a_{2}\right)\in A^{2}$ such that $P_{Y|X}\left(\cdot |a_{1}\right)⊥P_{Y|X}\left(\cdot |a_{2}\right)$.
21. The related quantity $\max_{P_{X}}I_{\frac{1}{\alpha }}\left(P_{X},P_{Y|X}^{\alpha }\right)$ arises in the study of the fundamental limits of guessing and task completion under mismatch [41,42].
22. While D(P∥Q) is *convex* in the pair (P,Q), the picture for Rényi divergence is somewhat more nuanced:
  (a) If α∈(0,1), then $D_{\alpha }\left(P\;∥\;Q\right)$ is convex in (P,Q).
  (b) If α>0, then $D_{\alpha }\left(P\;∥\;Q\right)$ is convex in *Q* for all *P*, (see [4]).
23. For any fixed pair $\left(P_{Y|X},Q_{Y|X}\right)$, $D_{\alpha }(P_{Y|X}∥Q_{Y|X}\left|P_{X}\right)$ is concave (resp. convex) in $P_{X}$ if α≥1 (resp. α∈(0,1]) (see [43]).
24. The α-mutual information $I_{\alpha }\left(P_{X},P_{Y|X}\right)$ is concave in $P_{X}$ for any fixed $P_{Y|X}$ and α>1 (see [43]). If α∈(0,1)∪(1,∞), then the following monotonically increasing function of $I_{\alpha }\left(P_{X},P_{Y|X}\right)$ is concave in $P_{X}$
  (57) $$\begin{array}{c} \Gamma_{\alpha }\left(I_{\alpha }\left(P_{X},P_{Y|X}\right)\right)=\frac{1}{\alpha -1}\varphi_{\frac{1}{\alpha }}\left(I_{\alpha }\left(P_{X},P_{Y|X}\right)\right), \end{array}$$
  where $\varphi_{\alpha }\left(z\right)=\exp\left(z-\alpha z\right)$ (see [10,43]).

## 3. Conditional Rényi Divergence Game

As can be expected from (43), when maximizing α-mutual information, for fixed $P_{Y|X}$, with respect to the input probability measure, it is interesting to consider a zero-sum game with payoff function

$$D_{\alpha }(P_{Y|X}\;∥\;Q|P_{X})$$

such that one player tries to maximize it by choosing $P_{X}\in P$, where P is a given collection of input probability measures, and the other player tries to minimize it by choosing the probability measure Q∈Q on the output space. Balancing simplicity and generality and motivated by applications, while we allow P to be a proper subset of the set of all input probability measures, we assume that there are no restrictions in the choice of the output probability measure, and therefore Q stands for the whole collection of probability measures on the output space. This game also arises in the determination of the worst-case redundancy in (37). In Section 3.1 we consider the important special case in which there exists an input distribution that attains the supremum in (55). In the more general scenario in which the supremum may not be achieved, we cannot identify a saddle point but we can indeed swap sup and min as we show in Section 3.2.

### 3.1. Saddle point

We begin by showing that the maximal α-mutual information input distribution and its α-response form a saddle point.

**Theorem** **1.** *Let P be a convex set of probability distributions on A and Q be the set of all probability distributions on B. Let α∈(0,1)∪(1,∞). Suppose that there exists some $P_{X}^{*}\in P$ such that*

(58) $$\begin{array}{c} I_{\alpha }\left(P_{X}^{*},P_{Y|X}\right)=\max_{P_{X}\in P}I_{\alpha }\left(P_{X},P_{Y|X}\right)<\infty , \end{array}$$

*and denote the α-response to $P_{X}^{*}$ by $P_{Y_{\left[\alpha \right]}}^{*}$. Then, for any $(P_{X},Q_{Y})\in P\times Q$,*

(59) $$\begin{array}{cc} D_{\alpha }(P_{Y|X}∥P_{Y_{\left[\alpha \right]}}^{*}\left|P_{X}\right) & \leq D_{\alpha }(P_{Y|X}∥P_{Y_{\left[\alpha \right]}}^{*}\left|P_{X}^{*}\right) \end{array}$$

(60) $$\begin{array}{cc} \; & \leq D_{\alpha }(P_{Y|X}∥Q_{Y}\left|P_{X}^{*}\right). \end{array}$$

*Conversely, if $\left(P_{X}^{*},P_{Y_{\left[\alpha \right]}}^{*}\right)$ is a saddle point of $D_{\alpha }(P_{Y|X}∥\cdot |\cdot )$, namely, (59)–(60) are satisfied, then $P_{X}^{*}$ maximizes the α-mutual information.*

**Remark** **1.** *Assuming that P includes $\delta_{x}$ (unit mass at x∈A), (59) implies that for any x∈A,*

(61) $$\begin{array}{c} D_{\alpha }\left(P_{Y|X=x}∥P_{Y_{\left[\alpha \right]}}^{*}\right)\leq \max_{P_{X}\in P}I_{\alpha }\left(P_{X},P_{Y|X}\right). \end{array}$$

We can easily obtain corollaries to Theorem 1 that elucidate useful properties of the saddle point.

**Corollary** **1.** *Let α∈(0,1)∪(1,∞). Under the assumptions in Theorem 1, for any $P_{X}\in P$, we have*

(62) $$\begin{array}{c} D_{\alpha }\left(P_{Y_{\left[\alpha \right]}}∥P_{Y_{\left[\alpha \right]}}^{*}\right)\leq I_{\alpha }\left(P_{X}^{*},P_{Y|X}\right)-I_{\alpha }\left(P_{X},P_{Y|X}\right)<\infty , \end{array}$$

*where $P_{Y_{\left[\alpha \right]}}$ is the α-response to $P_{X}$. Moreover, $P_{Y_{\left[\alpha \right]}}=P_{Y_{\left[\alpha \right]}}^{*}$ if, in addition to $P_{X}^{*}$, $P_{X}$ also attains $C_{\alpha }\left(P\right)=\max_{P_{X}\in P}I_{\alpha }\left(P_{X},P_{Y|X}\right)$.*

**Proof of Corollary** **1.** For any $P_{X}\in P$,

(63) $$\begin{array}{cc} I_{\alpha }\left(P_{X},P_{Y|X}\right) & =D_{\alpha }(P_{Y|X}∥P_{Y_{\left[\alpha \right]}}\left|P_{X}\right) \end{array}$$

(64) $$\begin{array}{cc} \; & =D_{\alpha }(P_{Y|X}∥P_{Y_{\left[\alpha \right]}}^{*}\left|P_{X}\right)-D_{\alpha }\left(P_{Y_{\left[\alpha \right]}}∥P_{Y_{\left[\alpha \right]}}^{*}\right) \end{array}$$

(65) $$\begin{array}{cc} \; & \leq D_{\alpha }(P_{Y|X}∥P_{Y_{\left[\alpha \right]}}^{*}\left|P_{X}^{*}\right)-D_{\alpha }\left(P_{Y_{\left[\alpha \right]}}∥P_{Y_{\left[\alpha \right]}}^{*}\right) \end{array}$$

(66) $$\begin{array}{cc} \; & =I_{\alpha }\left(P_{X}^{*},P_{Y|X}\right)-D_{\alpha }\left(P_{Y_{\left[\alpha \right]}}∥P_{Y_{\left[\alpha \right]}}^{*}\right), \end{array}$$

where (64) and (65) follow from (42) and (59), respectively. Since Rényi divergence is nonnegative, $D_{\alpha }\left(P_{Y_{\left[\alpha \right]}}∥P_{Y_{\left[\alpha \right]}}^{*}\right)=0$ if $P_{X}$ also attains $C_{\alpha }\left(P\right)$.  □

Therefore, Corollary 1 implies that the α-responses to all the maximal α-mutual information input distributions must be identical. Moreover, if α>1, then every α-response to any input distribution satisfies $P_{Y_{\left[\alpha \right]}}\ll P_{Y_{\left[\alpha \right]}}^{*}$.

If P is the space of all probability distributions on A, then we can get the following corollary.

**Corollary** **2.** *Unconstrained maximization of α-mutual information. Suppose that α∈(0,1)∪(1,∞) and P contains all probability mass functions on the discrete alphabet A. Fix $P_{Y|X}:A\to B$. For any input distribution ${\bar{P}}_{X}$, denote its support by ${\bar{A}}_{X}\subset A$ and the corresponding α-response by ${\bar{P}}_{Y_{\left[\alpha \right]}}$.*

*A necessary and sufficient condition for ${\bar{P}}_{X}$ to achieve $\max_{X}I\left(X;Y\right)<\infty$ is*

(67) $$\begin{array}{c} \max_{a\in {\bar{A}}_{X}}D_{\alpha }\left(P_{Y|X=a}∥{\bar{P}}_{Y_{\left[\alpha \right]}}\right)=\min_{a\in {\bar{A}}_{X}}D_{\alpha }\left(P_{Y|X=a}∥{\bar{P}}_{Y_{\left[\alpha \right]}}\right)\geq \max_{a\in {\bar{A}}_{X}^{c}}D_{\alpha }\left(P_{Y|X=a}∥{\bar{P}}_{Y_{\left[\alpha \right]}}\right). \end{array}$$

**Proof of Corollary** **2.**

- $\max_{X}I_{\alpha }\left(X;Y\right)=I_{\alpha }\left({\bar{P}}_{X},P_{Y|X}\right)\Rightarrow \left(67\right)$: Regardless of whether α>1 or α<1, we see from (45) that if there exists some $x_{0}\in {\bar{A}}_{X}$ such that
  (68) $$\begin{array}{c} D_{\alpha }\left(P_{Y|X=x_{0}}∥{\bar{P}}_{Y_{\left[\alpha \right]}}\right)<\max_{P_{X}\in P}I_{\alpha }\left(P_{X},P_{Y|X}\right), \end{array}$$
  then $I_{\alpha }\left({\bar{P}}_{X},P_{Y|X}\right)<\max_{P_{X}\in P}I_{\alpha }\left(P_{X},P_{Y|X}\right)$, which contradicts the assumed optimality of ${\bar{P}}_{X}$. Moreover, if there exists some $x_{0}\in {\bar{A}}_{X}^{c}$ such that (68) holds with the strict inequality reversed, then (59) would be violated, again contradicting the assumed optimality of ${\bar{P}}_{X}$.
- $\left(67\right)\Rightarrow \max_{X}I_{\alpha }\left(X;Y\right)=I_{\alpha }\left({\bar{P}}_{X},P_{Y|X}\right)$: Again, we see from (45) that if (67) is satisfied, then (59) is satisfied. Since ${\bar{P}}_{Y_{\left[\alpha \right]}}$ is the α-response to ${\bar{P}}_{X}$, (59) is also satisfied, and the converse part in Theorem 1 results in the optimality of ${\bar{P}}_{X}$.

 □

**Remark** **2.** *According to Corollary 2, if some input distribution $P_{X}^{*}$ achieves $C_{\alpha }$, we know the α-response output distribution $P_{Y_{\left[\alpha \right]}}^{*}$ is equidistant in $D_{\alpha }\left(\cdot ∥P_{Y_{\left[\alpha \right]}}^{*}\right)$ to any of the output distributions in the collection*

(69) $$\begin{array}{c} S=\{P_{Y|X=x},\;P_{X}^{*}\left(x\right)>0\}\subset Q. \end{array}$$

*Moreover, we know that the optimal α-response output distribution is actually unique even if there exist several optimal input distributions. So the key is to find the unique centroid of S when the distance is measured by the Rényi divergence. In contrast to the maximization of the mutual information, the optimal α-response output distribution is no longer a mixture of the conditional output distributions.*

**Remark** **3.** *Corollary 2 enables us to recover Gallager’s finite alphabet result in Theorem 5.6.5 of [44], which characterizes the maximal α-mutual information input distribution if α∈(0,1) when both A and B are finite. The optimal input distribution $P_{X}^{*}$ must satisfy the following condition:*

(70) $$\begin{array}{c} \sum_{y\in B}{\left(\sum_{x\in A}P_{Y|X}^{\alpha }\left(y|x\right)P_{X}^{*}\left(x\right)\right)}^{\frac{1-\alpha }{\alpha }}P_{Y|X}^{\alpha }\left(y|u\right)\geq \sum_{y\in B}{\left(\sum_{x\in A}P_{Y|X}^{\alpha }\left(y|x\right)P_{X}^{*}\left(x\right)\right)}^{\frac{1}{\alpha }}, \end{array}$$

*with equality for all u such that $P_{X}^{*}\left(u\right)>0$. To verify this condition, note that Corollary 2 requires that*

(71) $$\begin{array}{c} \exp\left(\kappa_{\alpha }^{*}\right)=\exp\left(\left(\alpha -1\right)C_{\alpha }\right)\leq \sum_{y\in B}P_{Y|X=u}^{\alpha }\left(y\right){\left(P_{Y_{\left[\alpha \right]}}^{*}\left(y\right)\right)}^{1-\alpha }, \end{array}$$

*with equality if $P_{X}^{*}\left(u\right)>0$, and where $\kappa_{\alpha }^{*}$ stands for the normalizing constant in (24) with $P_{X}\leftarrow P_{X}^{*}$. Upon substitution of (25) with $P_{X}\leftarrow P_{X}^{*}$, we obtain (70). The assumption of finite output alphabet can be easily dispensed with to obtain the more general optimality condition*

(72) $$\begin{array}{c} E\left[\Psi_{\alpha }^{1-\alpha }\left(Y^{*}\right)\exp\left(\alpha \;{ı}_{X;Y}^{*}\left(u;Y^{*}\right)\right)\right]\geq E\left[\Psi_{\alpha }\left(Y^{*}\right)\right] \end{array}$$

*with equality for all u such that $P_{X}^{*}\left(u\right)>0$. In (72), ${ı}_{X;Y}^{*}$ stands for the information density corresponding to $P_{X}^{*}\to P_{Y|X}\to P_{Y}^{*}$ and*

(73) $$\begin{array}{c} \Psi_{\alpha }\left(y\right)=E^{\frac{1}{\alpha }}\left[\exp\left(\alpha \;{ı}_{X;Y}^{*}\left(X^{*};y\right)\right)\right],\;X^{*}∼P_{X}^{*} \end{array}$$

If α>1, condition (72) holds with the inequality reversed.

**Remark** **4.** *When B is finite, it was shown in [2,4,25] that for any α∈[0,∞],*

(74) $$\begin{array}{c} C_{\alpha }=R_{\alpha }, \end{array}$$

*where $R_{\alpha }$ is defined in (37). This is now established without imposing finiteness conditions, as long as there is an input that achieves the maximal α-mutual information because*

(75) $$\begin{array}{cc} R_{\alpha } & \leq C_{\alpha } \end{array}$$

(76) $$\begin{array}{cc} \; & =\max_{P_{X}\in P}\min_{Q_{Y}\in Q}D_{\alpha }(P_{Y|X}∥Q_{Y}\left|P_{X}\right) \end{array}$$

(77) $$\begin{array}{cc} \; & \leq \min_{Q_{Y}\in Q}\max_{P_{X}\in P}D_{\alpha }(P_{Y|X}∥Q_{Y}\left|P_{X}\right) \end{array}$$

(78) $$\begin{array}{cc} \; & \leq R_{\alpha }, \end{array}$$

*where (75) follows from particularizing (59) to deterministic $P_{X}$, and () follows from (40).*

### 3.2. Minimax identity

In this section we drop the assumption that there exists an input probability measure that attains the maximal α-mutual information and show that the conditional Rényi divergence still satisfies a minimax identity, even if a saddle point does not exist.

**Theorem** **2.** *Let P be a convex set of probability distributions on A and Q be the set of all probability distributions on B. We have the minimax equality:*

(79) $$\begin{array}{cc} C_{\alpha }\left(P\right)\; & =\underset{P_{X}\in P}{\sup}\min_{Q_{Y}\in Q}D_{\alpha }(P_{Y|X}∥Q_{Y}\left|P_{X}\right) \end{array}$$

(80) $$\begin{array}{cc} \; & =\min_{Q_{Y}\in Q}\underset{P_{X}\in P}{\sup}D_{\alpha }(P_{Y|X}∥Q_{Y}\left|P_{X}\right). \end{array}$$

*Furthermore, if $C_{\alpha }\left(P\right)<\infty$, then there exists a unique element in Q attaining the minimum in (80).*

The assumption of convexity in Theorem 2 is not superfluous, as the following example illustrates.

**Example** **1.** *Let A=B=N and Y=X+N, where N is a geometric random variable on the nonnegative integers with positive mean and independent of X. Let P be the non-convex set of all the deterministic probability measures on A. In this case, the left side of (80) is zero, while the right side is infinity. To see this, note that for any $Q_{Y}\in Q$ and n∈N, it follows from the data processing inequality applied to the binary deterministic transformation 1{Y≥n} that*

(81) $$\begin{array}{c} D_{\alpha }\left(P_{Y|X=n}∥Q_{Y}\right)\geq d_{\alpha }\left(1∥Q_{Y}\left(\left\{n+1,\ldots \right\}\right)\right), \end{array}$$

*whose right side diverges as n→∞. Therefore, for any $Q_{Y}\in Q$, (39) results in*

(82) $$\begin{array}{c} \underset{P_{X}\in P}{\sup}D_{\alpha }(P_{Y|X}∥Q_{Y}\left|P_{X}\right)=\infty . \end{array}$$

Continuing with the theme in Remark 4, Theorem 2 extends the validity of $R_{\alpha }=C_{\alpha }$ without requiring the existence of the maximal α-mutual information input distribution. It was conjectured in [2] (and proved in [26]) that for α∈(0,∞), if $R_{\alpha }<\infty$ and B is finite or countable, there exists a unique redundancy-achieving distribution

(83) $$\begin{array}{c} Q_{Y}^{*}=\arg\min_{Q_{Y}}\underset{x\in A}{\sup}D_{\alpha }\left(P_{Y|X=x}∥Q_{Y}\right) \end{array}$$

and for all probability measures $Q_{Y}$ on the output space,

(84) $$\begin{array}{c} \underset{x\in A}{\sup}D_{\alpha }\left(P_{Y|X=x}∥Q_{Y}\right)\geq C_{\alpha }+D_{\alpha }\left(Q_{Y}^{*}∥Q_{Y}\right). \end{array}$$

We can prove the conjecture easily with the help of Theorem 2.

**Proof.**  Let P be the convex set of all probability measures on A. Since $C_{\alpha }=R_{\alpha }<\infty$, by Theorem 2, we know there exists a unique $P_{Y_{\left[\alpha \right]}}^{*}$ such that $\sup_{P_{X}\in P}D_{\alpha }(P_{Y|X}∥P_{Y_{\left[\alpha \right]}}^{*}\left|P_{X}\right)=C_{\alpha }$, which implies that $P_{Y_{\left[\alpha \right]}}^{*}$ is precisely the unique redundancy-achieving distribution in (83). Moreover, as shown in the proof of Theorem 2, we can find a sequence ${\left\{P_{X_{n}}\right\}}_{n\geq 1}$ in P such that $I_{\alpha }\left(P_{X_{n}},P_{Y|X}\right)\to C_{\alpha }$ as n→∞ and such that the corresponding α-responses $P_{Y_{n[\alpha ]}}$ converge to $P_{Y_{\left[\alpha \right]}}^{*}$ in the total variation metric. Pick an arbitrary $Q_{Y}\in Q$. If α>1 and PY|X=x≪QY for some x∈A, then $\sup_{x\in A}D_{\alpha }\left(P_{Y|X=x}∥Q_{Y}\right)=\infty$ and (84) holds. Otherwise, by (42) we always have

(85) $$\begin{array}{c} D_{\alpha }(P_{Y|X}∥Q_{Y}|P_{X})=D_{\alpha }(P_{Y|X}∥P_{Y_{\left[\alpha \right]}}^{*}\left|P_{X}\right)+D_{\alpha }\left(P_{Y_{\left[\alpha \right]}}^{*}∥Q_{Y}\right). \end{array}$$

For any n≥1, since P includes all probability measures on A, we have

(86) $$\begin{array}{cc} \underset{x\in A}{\sup}D_{\alpha }\left(P_{Y|X=x}∥Q_{Y}\right) & =\underset{P_{X}\in P}{\sup}D_{\alpha }(P_{Y|X}∥Q_{Y}\left|P_{X}\right) \end{array}$$

(87) $$\begin{array}{cc} \; & \geq D_{\alpha }(P_{Y|X}∥Q_{Y}\left|P_{X_{n}}\right) \end{array}$$

(88) $$\begin{array}{cc} \; & =D_{\alpha }(P_{Y|X}∥P_{Y_{n[\alpha ]}}\left|P_{X_{n}}\right)+D_{\alpha }\left(P_{Y_{n[\alpha ]}}∥Q_{Y}\right) \end{array}$$

(89) $$\begin{array}{cc} \; & =I_{\alpha }\left(P_{X_{n}},P_{Y|X}\right)+D_{\alpha }\left(P_{Y_{n[\alpha ]}}∥Q_{Y}\right), \end{array}$$

where (88) is due to (42). Taking the limit as n→∞, the lower-semicontinuity of the Rényi divergence ensures that

(90) $$\begin{array}{c} \underset{x\in A}{\sup}D_{\alpha }\left(P_{Y|X=x}∥Q_{Y}\right)\geq C_{\alpha }+D_{\alpha }\left(P_{Y_{\left[\alpha \right]}}^{*}∥Q_{Y}\right), \end{array}$$

and therefore the sought-after $Q_{Y}^{*}$ is none other that $P_{Y_{\left[\alpha \right]}}^{*}$, the unique maximal α-mutual information output distribution.  □

## 4. Finding $C_{\alpha }$

In this section, we present a number of examples to illustrate how the results in Section 3 can be used to maximize the α-mutual information with respect to the input distribution. It is instructive to contrast the present approach with the maximization of α-mutual information invoking the KKT conditions, which is feasible in both the case α>1 in which the functional is concave with respect to the input distribution, and the case α∈(0,1) in which a monotonically increasing function of α-mutual information is concave. Simple finite-alphabet examples of such approach can be found in [44] when dealing with the $E_{0}$ functional in (54). Thanks to Theorem 1 it is possible to avoid taking derivatives of any functionals.

**Example 2** (Binary symmetric channel)**.** *Let the input and output alphabet be A=B={0,1} and the random transformation be*

(91) $$\begin{array}{c} P_{Y|X}=\left[\begin{array}{cc} 1-\delta & \delta \\ \delta & 1-\delta \end{array}\right],\;\delta \in \left[0,1\right]. \end{array}$$

*Let’s try the input distribution $P_{X}^{*}\left(0\right)=P_{X}^{*}\left(1\right)=0.5$. Then, according to (25), the α-response output distribution is also equiprobable $P_{Y_{\left[\alpha \right]}}^{*}\left(0\right)=P_{Y_{\left[\alpha \right]}}^{*}\left(1\right)=0.5$. Since*

(92) $$\begin{array}{cc} D_{\alpha }\left(P_{Y|X=0}∥P_{Y_{\left[\alpha \right]}}^{*}\right) & =D_{\alpha }\left(P_{Y|X=1}∥P_{Y_{\left[\alpha \right]}}^{*}\right)=d_{\alpha }\left(\delta \;∥\frac{1}{2}\right) \end{array}$$

*the conditions of Corollary 2 are met, $P_{X}^{*}$ attains the maximal α-mutual information and therefore,*

(93) $$\begin{array}{c} C_{\alpha }=d_{\alpha }\left(\delta \;∥\frac{1}{2}\right)=\log2+\frac{1}{\alpha -1}\log\left(\delta^{\alpha }+{\left(1-\delta \right)}^{\alpha }\right), \end{array}$$

*which satisfies, according to (31)*

(94) $$\begin{array}{cc} \lim_{\alpha \to 1}C_{\alpha } & =\log2-h(\alpha ), \end{array}$$

(95) $$\begin{array}{cc} \lim_{\alpha \to \infty }C_{\alpha } & =\log\left(2\max\{\delta ,1-\delta \}\right). \end{array}$$

**Example 3** (Binary erasure channel.)**.** *Let the input/output alphabets be A={0,1} and B={0,e,1}, and the random transformation be*

(96) $$\begin{array}{c} P_{Y|X}=\left[\begin{array}{cc} 1-\delta & 0\\ \delta & \delta \\ 0 & 1-\delta \end{array}\right],\;\delta \in \left[0,1\right]. \end{array}$$

*(Departing from usual practice, columns/rows represent input/output letters respectively, i.e., probability vectors are column vectors, although for typographical convenience we show them as row vectors in the text.)*

*The α-response output distribution to $P_{X}^{*}\left(0\right)=P_{X}^{*}\left(1\right)=0.5$ is*

(97) $$\begin{array}{cc} P_{Y_{\left[\alpha \right]}}^{*}\left(0\right)=P_{Y_{\left[\alpha \right]}}^{*}\left(1\right) & =\frac{1-\delta }{\delta 2^{\frac{1}{\alpha }}+2\left(1-\delta \right)}, \end{array}$$

(98) $$\begin{array}{cc} P_{Y_{\left[\alpha \right]}}^{*}\left(e\right) & =\frac{\delta 2^{\frac{1}{\alpha }}}{\delta 2^{\frac{1}{\alpha }}+2\left(1-\delta \right)}. \end{array}$$

*By symmetry,*

(99) $$\begin{array}{cc} C_{\alpha } & =D_{\alpha }\left(P_{Y|X=0}∥P_{Y_{\left[\alpha \right]}}^{*}\right)=D_{\alpha }\left(P_{Y|X=1}∥P_{Y_{\left[\alpha \right]}}^{*}\right) \end{array}$$

(100) $$\begin{array}{cc} \; & =\frac{1}{\alpha -1}\log\left({\left(1-\delta \right)}^{\alpha }{\left(\frac{\left(1-\delta \right)}{\delta 2^{\frac{1}{\alpha }}+2\left(1-\delta \right)}\right)}^{1-\alpha }+\delta^{\alpha }{\left(\frac{\delta 2^{\frac{1}{\alpha }}}{\delta 2^{\frac{1}{\alpha }}+2\left(1-\delta \right)}\right)}^{1-\alpha }\right) \end{array}$$

(101) $$\begin{array}{c} =\frac{1}{\alpha -1}\log\left(\frac{1-\delta +2^{\frac{1-\alpha }{\alpha }}\delta }{{\left(\delta 2^{\frac{1}{\alpha }}+2\left(1-\delta \right)\right)}^{1-\alpha }}\right) \end{array}$$

(102) $$\begin{array}{c} =\frac{1}{\alpha -1}\log\left(\frac{2^{\frac{\alpha -1}{\alpha }}\left(1-\delta \right)+\delta }{{\left(\delta +2^{\frac{\alpha -1}{\alpha }}\left(1-\delta \right)\right)}^{1-\alpha }}\right) \end{array}$$

(103) $$\begin{array}{c} =\frac{\alpha }{\alpha -1}\log\left(\left(1-\delta \right)2^{\left(1-\frac{1}{\alpha }\right)}+\delta \right), \end{array}$$

*which satisfies (in bits)*

(104) $$\begin{array}{cc} \lim_{\alpha \to 1}C_{\alpha } & =1-\delta , \end{array}$$

(105) $$\begin{array}{cc} \lim_{\alpha \to \infty }C_{\alpha } & =\log_{2}\left(2-\delta \right). \end{array}$$

**Example 4** (Binary asymmetric channel)**.** *Let the input and output alphabet be A=B={0,1} and the random transformation be*

(106) $$\begin{array}{c} P_{Y|X}=\left[\begin{array}{cc} 1-\delta_{0} & \delta_{1}\\ \delta_{0} & 1-\delta_{1} \end{array}\right],\;\left(\delta_{0},\delta_{1}\right)\in {\left[0,1\right]}^{2}. \end{array}$$

*If $\delta_{0}+\delta_{1}=1$, then $I_{\alpha }\left(X;Y\right)=0$ for any input distribution. We will assume $\delta_{0}+\delta_{1}<1$. Otherwise, we can just relabel the output alphabet (0,1)←(1,0), or equivalently $\left(\delta_{0},\delta_{1}\right)\leftarrow \left(1-\delta_{0},1-\delta_{1}\right)$. The condition*

(107) $$\begin{array}{c} D_{\alpha }\left(P_{Y|X=0}∥P_{Y_{\left[\alpha \right]}}^{*}\right)=D_{\alpha }\left(P_{Y|X=1}∥P_{Y_{\left[\alpha \right]}}^{*}\right) \end{array}$$

*is now $d_{\alpha }\left(1-\delta_{0}∥P_{Y_{\left[\alpha \right]}}^{*}\left(0\right)\right)=d_{\alpha }\left(\delta_{1}∥P_{Y_{\left[\alpha \right]}}^{*}\left(0\right)\right)$, which, in view of (32) yields*

(108) $$\begin{array}{c} P_{Y_{\left[\alpha \right]}}^{*}\left(0\right)={\left(1+{\left(\frac{{\left(1-\delta_{0}\right)}^{\alpha }-\delta_{1}^{\alpha }}{{\left(1-\delta_{1}\right)}^{\alpha }-\delta_{0}^{\alpha }}\right)}^{\frac{1}{1-\alpha }}\right)}^{-1}. \end{array}$$

*We can verify from (25) that this corresponds to the α-response to $P_{X}^{*}\left(1\right)=p^{*}=1-P_{X}^{*}\left(0\right)$, where $p^{*}\in \left[0,1\right]$ is the solution to*

(109) $$\begin{array}{c} \frac{\delta_{0}^{\alpha }\left(1-p\right)+{\left(1-\delta_{1}\right)}^{\alpha }p}{{\left(1-\delta_{0}\right)}^{\alpha }\left(1-p\right)+\delta_{1}^{\alpha }p}={\left(\frac{{\left(1-\delta_{0}\right)}^{\alpha }-\delta_{1}^{\alpha }}{{\left(1-\delta_{1}\right)}^{\alpha }-\delta_{0}^{\alpha }}\right)}^{\frac{\alpha }{1-\alpha }}. \end{array}$$

*Then,*

(110) $$\begin{array}{cc} C_{\alpha } & =D_{\alpha }\left(P_{Y|X=0}∥P_{Y_{\left[\alpha \right]}}^{*}\right) \end{array}$$

(111) $$\begin{array}{cc} \; & =\frac{1}{\alpha -1}\log\left({\left(1-\delta_{0}\right)}^{\alpha }{\left(1-\delta_{1}\right)}^{\alpha }-\delta_{0}^{\alpha }\delta_{1}^{\alpha }\right)+\log\left({\left({\left(1-\delta_{0}\right)}^{\alpha }-\delta_{1}^{\alpha }\right)}^{\frac{1}{1-\alpha }}+{\left({\left(1-\delta_{1}\right)}^{\alpha }-\delta_{0}^{\alpha }\right)}^{\frac{1}{1-\alpha }}\right). \end{array}$$

*which satisfies*

(112) $$\begin{array}{cc} \lim_{\alpha \to 1}C_{\alpha } & =\log\left(\exp\left(\frac{h(\delta_{0})}{1-\delta_{1}-\delta_{0}}\right)+\exp\left(\frac{h(\delta_{1})}{1-\delta_{1}-\delta_{0}}\right)\right)-\frac{\left(1-\delta_{1}\right)h\left(\delta_{0}\right)+\left(1-\delta_{0}\right)h\left(\delta_{1}\right)}{1-\delta_{1}-\delta_{0}}, \end{array}$$

*and*

(113) $$\begin{array}{cc} \lim_{\alpha \to \infty }C_{\alpha } & =\log(2-\delta_{0}-\delta_{1}). \end{array}$$

**Example 5** (Z channel)**.** *Let the input and output alphabet be A=B={0,1} and the random transformation be*

(114) $$\begin{array}{c} P_{Y|X}=\left[\begin{array}{cc} 1 & \delta \\ 0 & 1-\delta \end{array}\right],\;\delta \in \left[0,1\right]. \end{array}$$

*Since this is the special case $\left(\delta_{0},\delta_{1}\right)=\left(0,\delta \right)$ of the binary asymmetric channel we obtain*

(115) $$\begin{array}{c} C_{\alpha }=\log\left(1+{\left(\frac{1-\delta^{\alpha }}{{\left(1-\delta \right)}^{\alpha }}\right)}^{\frac{1}{1-\alpha }}\right). \end{array}$$

*The limit*

(116) $$\begin{array}{c} \lim_{\alpha \to 1}C_{\alpha }=\log\left(1-\delta^{\frac{1}{1-\delta }}+\delta^{\frac{\delta }{1-\delta }}\right) \end{array}$$

*coincides with the capacity of the Z-channel originally derived in [45].*

The next example illustrates a case in which there are multiple optimal input distributions.

**Example** **6.** *Let A={0,1,2}, B={0,1,2,3} and the random transformation be*

(117) $$\begin{array}{c} P_{Y|X}=\left[\begin{array}{ccc} \frac{1}{2}-\delta & \delta & \frac{1}{2}-\delta \\ \delta & \frac{1}{2}-\delta & \delta \\ \delta & \frac{1}{2}-\delta & \delta \\ \frac{1}{2}-\delta & \delta & \frac{1}{2}-\delta \end{array}\right],\;\delta \in \left[0,\frac{1}{2}\right]. \end{array}$$

*Let $P_{X}^{0}=\left[\frac{1}{2}\;\;\;\frac{1}{2}\;\;\;0\right]$ and $P_{X}^{1}=\left[0\;\;\;\frac{1}{2}\;\;\;\frac{1}{2}\right]$. Its easy to verify that the corresponding α-responses are the equiprobable distribution on B. To verify that $P_{X}^{0}$ and $P_{X}^{1}$ attain the maximal α-mutual information, denote $P_{Y_{\left[\alpha \right]}}^{*}=\left[\frac{1}{4}\;\;\;\frac{1}{4}\;\;\;\frac{1}{4}\;\;\;\frac{1}{4}\right]$. For all x∈A, we have*

(118) $$\begin{array}{cc} D_{\alpha }\left(P_{Y|X=x}∥P_{Y_{\left[\alpha \right]}}^{*}\right) & =D_{\alpha }\left(\left[\delta \;\;\;\frac{1}{2}-\delta \;\;\;\frac{1}{2}-\delta \;\;\;\delta \right]∥\left[\frac{1}{4}\;\;\;\frac{1}{4}\;\;\;\frac{1}{4}\;\;\;\frac{1}{4}\right]\right) \end{array}$$

(119) $$\begin{array}{cc} \; & =\frac{2\alpha -1}{\alpha -1}\log2+\log\left(\delta^{\alpha }+{\left(\frac{1}{2}-\delta \right)}^{\alpha }\right) \end{array}$$

(120) $$\begin{array}{c} =C_{\alpha }, \end{array}$$

*where (120) follows from Corollary 2.*

In the next example $C_{\alpha }$ is constant in α.

**Example 7** (Additive phase noise)**.** *Let the input and output alphabet be A=B=[0,2π) and the random transformation be $Y=\left(X+N\right)\;\mathrm{mod}\;2\pi$, where N is independent of X and is uniform on the interval $\left[-\theta_{0},\theta_{0}\right]$ with $\theta_{0}\in \left(0,\pi \right]$. Suppose $P_{X}^{*}$ is uniform on [0,2π), it is easy to verify that $P_{Y_{\left[\alpha \right]}}^{*}$ is also uniform on [0,2π). Invoking (26), we obtain*

(121) $$\begin{array}{c} D_{\alpha }\left(P_{Y|X=x}∥P_{Y_{\left[\alpha \right]}}^{*}\right)=\log\frac{\pi }{\theta_{0}},\;x\in A, \end{array}$$

*which according to Theorem 1 must be equal to $C_{\alpha }$ attained by $P_{X}^{*}$.*

**Example 8** (Additive Gaussian noise)**.** *Let A=B=R, Y=X+N, where $N∼N(0,\sigma^{2})$ is independent of X. Fix α∈(0,1) and P>0. Suppose that the set, P, of allowable input probability measures on A consists of those that satisfy*

(122) $$\begin{array}{c} E\left[\exp_{e}\left(-\frac{\alpha \left(1-\alpha \right)X^{2}}{2\left(\alpha^{2}P+\sigma^{2}\right)}\right)\right]\geq \sqrt{\frac{\alpha^{2}P+\sigma^{2}}{\alpha P+\sigma^{2}}}. \end{array}$$

*By completing the square, it is easy to verify that $P_{X}^{*}∼N\left(0,P\right)$ satisfies (122) with equality. Furthermore, its α-response is $P_{Y_{\left[\alpha \right]}}^{*}∼N\left(0,\alpha P+\sigma^{2}\right)$. To show that $P_{X}^{*}$ does indeed attain $C_{\alpha }\left(P\right)$, first we compute*

(123) $$\begin{array}{c} D_{\alpha }\left(P_{Y|X=x}∥P_{Y_{\left[\alpha \right]}}^{*}\right)=\frac{1}{2}\log\left(1+\frac{\alpha P}{\sigma^{2}}\right)-\frac{1}{2\left(1-\alpha \right)}\log\left(\frac{\alpha P+\sigma^{2}}{\alpha^{2}P+\sigma^{2}}\right)+\frac{1}{2}\frac{\alpha x^{2}}{\alpha^{2}P+\sigma^{2}}\log e. \end{array}$$

*With (122) and (123), it is straightforward to see that if $P_{X}\in P$ then*

(124) $$\begin{array}{c} D_{\alpha }\left(P_{Y|X}∥P_{Y_{\left[\alpha \right]}}^{*}|P_{X}\right)\leq D_{\alpha }\left(P_{Y|X}∥P_{Y_{\left[\alpha \right]}}^{*}|P_{X}^{*}\right). \end{array}$$

*Consequently, Theorem 1 establishes that $P_{X}^{*}$ achieves the maximal α-mutual information, which using (39) is given by*

(125) $$\begin{array}{cc} C_{\alpha }\left(P\right) & =\frac{1}{2}\log\left(1+\frac{\alpha P}{\sigma^{2}}\right). \end{array}$$

## 5. Proofs

### 5.1. Proof of Theorem 1

We deal with the converse statement first. If (59)–(60) are satisfied then $\left(P_{X}^{*},P_{Y_{\left[\alpha \right]}}^{*}\right)$ is a saddle point, and therefore,

(126) $$\begin{array}{cc} I_{\alpha }\left(P_{X}^{*},P_{Y|X}\right) & =\max_{P_{X}\in P}\min_{Q_{Y}\in Q}D_{\alpha }(P_{Y|X}∥Q_{Y}\left|P_{X}\right) \end{array}$$

(127) $$\begin{array}{cc} \; & =\min_{Q_{Y}\in Q}\max_{P_{X}\in P}D_{\alpha }(P_{Y|X}∥Q_{Y}\left|P_{X}\right). \end{array}$$

which, by definition of α-mutual information, implies that $P_{X}^{*}$ attains $\max_{P_{X}\in P}I_{\alpha }\left(P_{X},P_{Y|X}\right)$. To show that the optimal input and its α-response must form a saddle point, first we deal with the case α>1, in which we can use the concavity of the conditional Rényi divergence. Choose arbitrary ν∈(0,1) and $P_{X}\in P$. Let $P_{\nu }=\nu P_{X}+\left(1-\nu \right)P_{X}^{*}$ and denote its α-response by

(128) $$\begin{array}{c} Q_{Y_{\left[\alpha \right]}}^{\left(\nu \right)}=\underset{Q_{Y}\in Q}{arg min}D_{\alpha }(P_{Y|X}∥Q_{Y}\left|P_{\nu }\right). \end{array}$$

Therefore, $Q_{Y_{\left[\alpha \right]}}^{\left(0\right)}=P_{Y_{\left[\alpha \right]}}^{*}$. We have

(129) $$\begin{array}{cc} I_{\alpha }\left(P_{X}^{*},P_{Y|X}\right) & \geq I_{\alpha }\left(P_{\nu },P_{Y|X}\right) \end{array}$$

(130) $$\begin{array}{cc} \; & =D_{\alpha }(P_{Y|X}∥Q_{Y_{\left[\alpha \right]}}^{\left(\nu \right)}\left|P_{\nu }\right) \end{array}$$

(131) $$\begin{array}{cc} \; & \geq \nu D_{\alpha }(P_{Y|X}∥Q_{Y_{\left[\alpha \right]}}^{\left(\nu \right)}|P_{X})+\left(1-\nu \right)D_{\alpha }(P_{Y|X}∥Q_{Y_{\left[\alpha \right]}}^{\left(\nu \right)}\left|P_{X}^{*}\right) \end{array}$$

(132) $$\begin{array}{cc} \; & \geq \nu D_{\alpha }(P_{Y|X}∥Q_{Y_{\left[\alpha \right]}}^{\left(\nu \right)}|P_{X})+\left(1-\nu \right)\min_{Q_{Y}\in Q}D_{\alpha }(P_{Y|X}∥Q_{Y}\left|P_{X}^{*}\right) \end{array}$$

(133) $$\begin{array}{cc} \; & =\nu D_{\alpha }(P_{Y|X}∥Q_{Y_{\left[\alpha \right]}}^{\left(\nu \right)}\left|P_{X}\right)+\left(1-\nu \right)I_{\alpha }\left(P_{X}^{*},P_{Y|X}\right), \end{array}$$

where (129) is due to the assumption of the optimality of $P_{X}^{*}$, (131) holds because $D_{\alpha }(P_{Y|X}∥Q_{Y}\left|P_{X}\right)$ is concave in $P_{X}$ for α>1 and (130) and (133) are due to the definition of α-mutual information.

It follows that

(134) $$\begin{array}{c} D_{\alpha }(P_{Y|X}∥Q_{Y_{\left[\alpha \right]}}^{\left(\nu \right)}|P_{X})\leq I_{\alpha }\left(P_{X}^{*},P_{Y|X}\right)=D_{\alpha }(P_{Y|X}∥P_{Y_{\left[\alpha \right]}}^{*}\left|P_{X}^{*}\right). \end{array}$$

Since $|Q_{Y_{\left[\alpha \right]}}^{\left(\nu \right)}-Q_{Y_{\left[\alpha \right]}}^{\left(0\right)}|\to 0$ as ν→0, the lower semicontinuity of Rényi divergence and (134) imply (59).

We now show the desired result for α∈(0,1). In this case, the method of proof is easy to adapt to the α>1 case but it is more cumbersome than the foregoing proof, which is able to capitalize on the concavity of the conditional Rényi divergence. The starting point is the expression in (52) which we write as

(135) $$\begin{array}{cc} I_{\alpha }\left(P_{X},P_{Y|X}\right) & =\frac{\alpha }{\alpha -1}\log f\left(p_{X}\right), \end{array}$$

(136) $$\begin{array}{cc} f(r) & =\int_{B}{\left(\int_{A}p_{Y|X}^{\alpha }\left(y|x\right)r\left(x\right)\;d\mu_{X}\left(x\right)\right)}^{\frac{1}{\alpha }}\;d\mu_{Y}\left(y\right), \end{array}$$

where we have defined the functional *f* on the convex cone $\bar{P}$ of nonnegative functions *r* on the input space such that $r\left(x\right)=\beta \frac{\;dP}{\;d\mu_{X}}\left(x\right)$ for some β≥0 and P∈P. Recall from (48) that when the argument is a density, then we have

(137) $$\begin{array}{c} f\left(p_{X}\right)=E\left[E^{\frac{1}{\alpha }}\left[\exp\left(\alpha \;{ı}_{X;\bar{Y}}\left(X;\bar{Y}\right)\right)|\bar{Y}\right]\right],\;\left(X,\bar{Y}\right)∼P_{X}\times P_{\bar{Y}}. \end{array}$$

By virtue of the convexity of ${\left(\cdot \right)}^{\frac{1}{\alpha }}$, *f* is a convex functional. Its directional (Gateaux) derivative is given by (note that the assumed finiteness of $I_{\alpha }\left(X;Y\right)$ allows swapping of differentiation and integration by means of the dominated convergence theorem)

(138) $$\begin{array}{cc} f^{\prime }\left(r;q\right) & =\frac{\;d}{\;d\delta }{f\left(r+\delta q\right)|}_{\delta =0} \end{array}$$

(139) $$\begin{array}{cc} \; & =\frac{1}{\alpha }\int_{B}{\left(\int_{A}p_{Y|X}^{\alpha }\left(y|x\right)r\left(x\right)\;d\mu_{X}\left(x\right)\right)}^{\frac{1-\alpha }{\alpha }}\left(\int_{A}p_{Y|X}^{\alpha }\left(y|x\right)q\left(x\right)\;d\mu_{X}\left(x\right)\right)\;d\mu_{Y}\left(y\right). \end{array}$$

Define the Lagrangian

(140) $$\begin{array}{c} L\left(r,\lambda \right)=f\left(r\right)-\lambda \left(\int_{A}r\left(x\right)\;d\mu_{X}\left(x\right)-1\right). \end{array}$$

Since *f* is convex and (135) is maximized by $P_{X}^{*}$ among all probability measures on the convex set P, there exists some $\lambda_{0}\geq 0$ such that

(141) $$\begin{array}{c} \max_{\lambda \geq 0}\min_{r}L\left(r,\lambda \right)=L\left(p_{X}^{*},\lambda_{0}\right), \end{array}$$

where the minimization is over the convex cone $\bar{P}$. It follows from standard convex optimization (e.g., see p. 227 in Reference [46]) that the Gateaux derivative of $L(\cdot ,\lambda_{0})$ at $p_{X}^{*}$ in the direction of any $q\in \bar{P}$ satisfies

(142) $$\begin{array}{cc} L^{\prime }\left(p_{X}^{*};q,\lambda_{0}\right) & \geq 0, \end{array}$$

with equality if $q=p_{X}^{*}$. Invoking (139), we obtain

(143) $$\begin{array}{cc} L^{\prime }\left(p_{X}^{*};q,\lambda_{0}\right)= & \frac{1}{\alpha }\int_{B}{\left(\int_{A}p_{Y|X}^{\alpha }\left(y|x\right)p_{X}^{*}\left(x\right)\;d\mu_{X}\left(x\right)\right)}^{\frac{1-\alpha }{\alpha }}\left(\int_{A}p_{Y|X}^{\alpha }\left(y|x\right)q\left(x\right)\;d\mu_{X}\left(x\right)\right)\;d\mu_{Y}\left(y\right)\\ \; & -\lambda_{0}\left(\int_{A}p_{X}^{*}\left(x\right)\;d\mu_{X}\left(x\right)-1\right). \end{array}$$

Specializing (142) and its condition for equality to $q\leftarrow p_{X}$ we obtain

(144) $$\begin{array}{c} L^{\prime }\left(p_{X}^{*};p_{X},\lambda_{0}\right)\geq L^{\prime }\left(p_{X}^{*};p_{X}^{*},\lambda_{0}\right) \end{array}$$

which, upon substitution of (143), becomes

(145) $$\begin{array}{c} f\left(p_{X}^{*}\right)\leq \int_{A}\int_{B}p_{Y|X}^{\alpha }\left(y|x\right){\left(\int_{A}p_{Y|X}^{\alpha }\left(y|x\right)p_{X}^{*}\left(x\right)\;d\mu_{X}\left(x\right)\right)}^{\frac{1-\alpha }{\alpha }}p_{X}\left(x\right)\;d\mu_{Y}\left(y\right)\;d\mu_{X}\left(x\right). \end{array}$$

Taking $\frac{1}{\alpha -1}\log\left(\cdot \right)$ of both sides of (145), invoking (25) and (47), the inequality is reversed and we obtain

(146) $$\begin{array}{c} D_{\alpha }(P_{Y|X}∥P_{Y_{\left[\alpha \right]}}^{*}\left|P_{X}\right)+\frac{1-\alpha }{\alpha }I_{\alpha }\left(X^{*};Y^{*}\right)\leq \frac{1}{\alpha }I_{\alpha }\left(X^{*};Y^{*}\right), \end{array}$$

which upon rearranging is the sought-after inequality (59).

### 5.2. Proof of Theorem 2

In order to show

(147) $$\begin{array}{c} \underset{Q_{Y}\in Q}{\inf}\underset{P_{X}\in P}{\sup}D_{\alpha }(P_{Y|X}∥Q_{Y}|P_{X})\leq \underset{P_{X}\in P}{\sup}\min_{Q_{Y}\in Q}D_{\alpha }(P_{Y|X}∥Q_{Y}\left|P_{X}\right)=C_{\alpha }\left(P\right), \end{array}$$

we construct $Q_{Y}^{*}\in Q$ such that

(148) $$\begin{array}{c} D_{\alpha }(P_{Y|X}∥Q_{Y}^{*}\left|P_{X}\right)\leq C_{\alpha }\left(P\right),\;\forall P_{X}\in P. \end{array}$$

Moreover, $Q_{Y}^{*}$ is indeed the minimizer in the leftmost side of (147) and we may replace the inf with min therein.

The construction of $Q_{Y}^{*}$ follows a Cauchy-sequence approach in the proof of Kemperman’s result in [47]. Let ${\left\{P_{X_{n}}\right\}}_{n\geq 1}$ be a sequence of probability distributions in P such that

(149) $$\begin{array}{c} \lim_{n\to \infty }I_{\alpha }\left(P_{X_{n}},P_{Y|X}\right)=C_{\alpha }\left(P\right). \end{array}$$

Fix an arbitrary $P_{X}\in P$ and let $P_{n}\subset P$ denote the convex hull of $\left\{P_{X},P_{X_{1}},\cdots ,P_{X_{n}}\right\}$, which is a compact set. Although $I_{\alpha }\left(\cdot ,P_{Y|X}\right)$ may not be concave for α∈(0,1), recall from (57) that the monotonically increasing function $\Gamma_{\alpha }$ is such that $\Gamma_{\alpha }\left(I_{\alpha }\left(\cdot ,P_{Y|X}\right)\right)$ is concave. So there exists some $P_{X_{n}}^{*}\in P_{n}$ that attains $C_{\alpha }\left(P_{n}\right)$. Thus for any n≥1,

(150) $$\begin{array}{c} I_{\alpha }\left(P_{X_{n}},P_{Y|X}\right)\leq I_{\alpha }\left(P_{X_{n}}^{*},P_{Y|X}\right)\leq C_{\alpha }\left(P\right) \end{array}$$

by the definition of $C_{\alpha }\left(P\right)$. The asymptotic optimality of the sequence $X_{n}$ implies that $I_{\alpha }\left(P_{X_{n}}^{*},P_{Y|X}\right)=C_{\alpha }\left(P_{n}\right)$ also converges to $C_{\alpha }\left(P\right)$.

Denote by $P_{Y_{n[\alpha ]}}^{*}$ the α-response to $P_{X_{n}}^{*}$. Then for any m≥n≥1, we have

(151) $$\begin{array}{cc} I_{\alpha }\left(P_{X_{n}}^{*},P_{Y|X}\right) & =D_{\alpha }(P_{Y|X}∥P_{Y_{n[\alpha ]}}^{*}\left|P_{X_{n}}^{*}\right) \end{array}$$

(152) $$\begin{array}{cc} \; & =D_{\alpha }(P_{Y|X}∥P_{Y_{m[\alpha ]}}^{*}\left|P_{X_{n}}^{*}\right)-D_{\alpha }\left(P_{Y_{n[\alpha ]}}^{*}∥P_{Y_{m[\alpha ]}}^{*}\right) \end{array}$$

(153) $$\begin{array}{cc} \; & \leq D_{\alpha }(P_{Y|X}∥P_{Y_{m[\alpha ]}}^{*}\left|P_{X_{m}}^{*}\right)-D_{\alpha }\left(P_{Y_{n[\alpha ]}}^{*}∥P_{Y_{m[\alpha ]}}^{*}\right) \end{array}$$

(154) $$\begin{array}{cc} \; & =I_{\alpha }\left(P_{X_{m}}^{*},P_{Y|X}\right)-D_{\alpha }\left(P_{Y_{n[\alpha ]}}^{*}∥P_{Y_{m[\alpha ]}}^{*}\right), \end{array}$$

where (151) and (154) are due to the definition of α-mutual information, (152) follows from (42), and (153) holds because of Theorem 1 applied to $P_{m}$ since $P_{X_{n}}^{*}\in P_{m}$ as $P_{n}\subset P_{m}$ for m≥n. Rearranging the end-to-end inequality in (151)–(154) results in

(155) $$\begin{array}{c} D_{\alpha }\left(P_{Y_{n[\alpha ]}}^{*}∥P_{Y_{m[\alpha ]}}^{*}\right)\leq I_{\alpha }\left(P_{X_{m}}^{*},P_{Y|X}\right)-I_{\alpha }\left(P_{X_{n}}^{*},P_{Y|X}\right). \end{array}$$

But since $I_{\alpha }\left(P_{X_{n}}^{*},P_{Y|X}\right)$ converges to $C_{\alpha }\left(P\right)$, it is a Cauchy sequence, i.e.

(156) $$\begin{array}{c} \left|I_{\alpha }\left(P_{X_{n}}^{*},P_{Y|X}\right)-I_{\alpha }\left(P_{X_{m}}^{*},P_{Y|X}\right)\right|\to 0,\;n,m\to \infty . \end{array}$$

Hence, (155) ensures that $P_{Y_{n[\alpha ]}}^{*}$ is also a Cauchy sequence in the sense that $D_{\alpha }\left(P_{Y_{n[\alpha ]}}^{*}∥P_{Y_{m[\alpha ]}}^{*}\right)\to 0$ as n,m→∞. By the generalized Pinkser’s inequality (35), $P_{Y_{n[\alpha ]}}^{*}$ is also a Cauchy sequence in total variation distance, i.e., $|P_{Y_{n[\alpha ]}}^{*}-P_{Y_{m[\alpha ]}}^{*}|\to 0$ as n,m→∞. Since the space of probability measures is complete in the total variation distance, ${\left\{P_{Y_{n[\alpha ]}}^{*}\right\}}_{n}$ must possess a limit point, which we denote by $P_{Y_{\left[\alpha \right]}}^{*}$.

Now, by Theorem 1 applied to $P_{n}$, we have

(157) $$\begin{array}{c} D_{\alpha }(P_{Y|X}∥P_{Y_{n[\alpha ]}}^{*}|P_{X})\leq D_{\alpha }(P_{Y|X}∥P_{Y_{n[\alpha ]}}^{*}\left|P_{X_{n}}^{*}\right)\leq C_{\alpha }\left(P\right), \end{array}$$

and since $D_{\alpha }\left(P∥Q\right)$ is lower-semicontinuous in (P,Q) for α>0, taking limits as n→∞ of (157), we obtain

(158) $$\begin{array}{c} D_{\alpha }(P_{Y|X}∥P_{Y_{\left[\alpha \right]}}^{*}\left|P_{X}\right)\leq C_{\alpha }\left(P\right). \end{array}$$

Next we show that (158) holds for all $P_{X}\in P$, in other words, the limit point $P_{Y_{\left[\alpha \right]}}^{*}$ does not depend on the initial choice of $P_{X}\in P$. Choose an arbitrary distribution $Q_{X}\in P,Q_{X}\neq P_{X}$, and introduce the following notation: $P_{n}^{\prime }$ is the convex hull of $\left\{Q_{X},P_{X},P_{X_{1}},\cdots ,P_{X_{n}}\right\}$; $Q_{X_{n}}^{*}$ is a maximizer of $I_{\alpha }\left(P_{X},P_{Y|X}\right)$ in $P_{n}^{\prime }$; its α-response is $Q_{Y_{n[\alpha ]}}^{*}$; and $Q_{Y_{\left[\alpha \right]}}^{*}$ is the limit of the sequence ${\left\{Q_{Y_{n[\alpha ]}}^{*}\right\}}_{n}$. Then we have

(159) $$\begin{array}{cc} D_{\alpha }\left(P_{Y_{n[\alpha ]}}^{*}∥Q_{Y_{n[\alpha ]}}^{*}\right) & =D_{\alpha }(P_{Y|X}∥Q_{Y_{n[\alpha ]}}^{*}|P_{X_{n}}^{*})-D_{\alpha }(P_{Y|X}∥P_{Y_{n[\alpha ]}}^{*}\left|P_{X_{n}}^{*}\right) \end{array}$$

(160) $$\begin{array}{cc} \; & \leq D_{\alpha }(P_{Y|X}∥Q_{Y_{n[\alpha ]}}^{*}|Q_{X_{n}}^{*})-D_{\alpha }(P_{Y|X}∥P_{Y_{n[\alpha ]}}^{*}\left|P_{X_{n}}^{*}\right) \end{array}$$

(161) $$\begin{array}{cc} \; & =I_{\alpha }\left(Q_{X_{n}}^{*},P_{Y|X}\right)-I_{\alpha }\left(P_{X_{n}}^{*},P_{Y|X}\right), \end{array}$$

where (159) holds because of (42); (160) is due to Theorem 1 applied to $P_{n}^{\prime }$ and the fact that $P_{X_{n}}^{*}\in P_{n}\subset P_{n}^{\prime }$ for any n≥1; and (161) is because of the definition of the α-mutual information.

The same argument that led to the conclusion that $I_{\alpha }\left(P_{X_{n}}^{*},P_{Y|X}\right)\to C_{\alpha }\left(P\right)$ establishes that $I_{\alpha }\left(Q_{X_{n}}^{*},P_{Y|X}\right)\to C_{\alpha }\left(P\right)$. Therefore, taking limits as n→∞ in (159)–(161) and applying the lower-semicontinuity of $D_{\alpha }\left(P∥Q\right)$ again, we obtain

(162) $$\begin{array}{c} D_{\alpha }\left(P_{Y_{\left[\alpha \right]}}^{*}∥Q_{Y_{\left[\alpha \right]}}^{*}\right)=0, \end{array}$$

and therefore $P_{Y_{\left[\alpha \right]}}^{*}=Q_{Y_{\left[\alpha \right]}}^{*}.$ Since the limiting output distribution is the same whether we use $P_{n}$ or $P_{n}^{\prime }$ and according to the latter the roles of $P_{X}$ and $Q_{X}$ are identical, we conclude that had we defined $P_{n}$ with $Q_{X}$ instead of $P_{X}$, we would have reached the same limiting output distribution and (158) holds for all $P_{X}\in P$. So we have constructed $Q_{Y}^{*}$ satisfying (148).

Finally, we show that $P_{Y_{\left[\alpha \right]}}^{*}$ is the only element that achieves

$$\underset{Q_{Y}\in Q}{\inf}\underset{P_{X}\in P}{\sup}D_{\alpha }(P_{Y|X}∥Q_{Y}\left|P_{X}\right).$$

Arguing by contradiction, suppose that there exists another $\hat{P_{Y}}$ such that

(163) $$\begin{array}{c} \underset{P_{X}\in P}{\sup}D_{\alpha }(P_{Y|X}∥\hat{P_{Y}}\left|P_{X}\right)=C_{\alpha }\left(P\right). \end{array}$$

As earlier in the proof, let ${\left\{P_{X_{n}}\in P\right\}}_{n}$ be a sequence satisfying (149), and denote the corresponding α-responses by $P_{Y_{n[\alpha ]}}$. Then, invoking (42) again we have

(164) $$\begin{array}{cc} D_{\alpha }(P_{Y|X}∥P_{Y_{n[\alpha ]}}\left|P_{X_{n}}\right)+D_{\alpha }\left(P_{Y_{n[\alpha ]}}∥\hat{P_{Y}}\right) & =D_{\alpha }(P_{Y|X}∥\hat{P_{Y}}\left|P_{X_{n}}\right) \end{array}$$

(165) $$\begin{array}{cc} \; & \leq C_{\alpha }\left(P\right)<\infty , \end{array}$$

where the inequality follows from (163). Using (149) we obtain

(166) $$\begin{array}{cc} D_{\alpha }\left(P_{Y_{n[\alpha ]}}∥\hat{P_{Y}}\right) & \leq C_{\alpha }\left(P\right)-D_{\alpha }(P_{Y|X}∥P_{Y_{n[\alpha ]}}\left|P_{X_{n}}\right) \end{array}$$

(167) $$\begin{array}{c} \to 0, \end{array}$$

and by (35), it follows that

(168) $$\begin{array}{c} |P_{Y_{n[\alpha ]}}-\hat{P_{Y}}|\to 0. \end{array}$$

Furthermore, we established above that

(169) $$\begin{array}{c} |P_{Y_{n[\alpha ]}}-P_{Y_{\left[\alpha \right]}}^{*}|\to 0. \end{array}$$

So, by the triangle inequality, we conclude that $\hat{P_{Y}}=P_{Y_{\left[\alpha \right]}}^{*}$.

## 6. Conclusions

The supremization of α-mutual information with respect to the input distribution plays an important role in various information theoretic settings, most notably in the error exponent of optimal codes operating below capacity. We show that the optimal (if it exists) input distribution, together with its α-response, form a saddle-point of the conditional Rényi divergence, and vice versa, the existence of the saddle point ensure the existence of a maximal α-mutual information input distribution. The application of this result to various discrete and non-discrete settings illustrates the power and generality of this tool, which mirrors a similar result enjoyed by conditional relative entropy; However, the proof of the latter result is much easier due to the more convenient structure of the objective function. Regardless of whether there exists an input distribution maximizing α-mutual information, there always exists a unique optimal output distribution, which is the limit of the α-responses of any asymptotically optimal sequence of input distributions. Furthermore, a saddle-value exists and

(170) $$\begin{array}{c} \underset{P_{X}}{\sup}\min_{Q}D_{\alpha }(P_{Y|X}\;∥\;Q|P_{X})=\min_{Q}\underset{P_{X}}{\sup}D_{\alpha }(P_{Y|X}\;∥\;Q|P_{X}) \end{array}$$

even if we restrict the feasible set of input distributions to be an arbitrary convex subset. These results lend further evidence to the notion that, out of all the available Rényi-generalizations of mutual information, the α-mutual information defined as in (43) is the most convenient and insightful, although $I_{\alpha }^{c}\left(X;Y\right)$ is also of considerable interest particularly in the error exponent analysis of channels with cost constraints.
